# Targeting of Kaposi’s sarcoma-associated herpesvirus by immunotoxins directed against the viral G protein-coupled receptor, ORF74

**DOI:** 10.1016/j.biopha.2025.118797

**Published:** 2026-02

**Authors:** Dagmar Fæster Kildedal, Anna Katarzyna Drzazga, Anjali Sharma, Christian Berg, Astrid Norup Winther, Laura Krogh-Hansen, Martin Gustavsson, Birthe B. Kragelund, Jon Våbenø, Michael Lagunoff, Naotaka Tsutsumi, Thomas N. Kledal, Mads G. Jeppesen, Mette M. Rosenkilde

**Affiliations:** a Molecular Pharmacology, Department of Biomedical Sciences, University of Copenhagen, Copenhagen, Denmark; b Synklino A/S, Copenhagen, Denmark; c Institute of Molecular and Industrial Biotechnology, Faculty of Biotechnology and Food Sciences, Lodz University of Technology, Lodz, Poland; d Department of Microbiology, University of Washington, Seattle, WA, USA; e Structural Biology and NMR Laboratory, Department of Biology, University of Copenhagen, Copenhagen, Denmark; f Helgeland Hospital Trust, Sandnessjøen, Norway; g Advanced Research Initiative, Institute of Integrated Research, Institute of Science Tokyo, Tokyo, Japan

**Keywords:** Kaposi Sarcoma-associated Herpesvirus (KSHV), Cancer, ORF74, Immunotoxins, Chemokine receptors

## Abstract

**Background and purpose:**

Kaposi’s sarcoma-associated herpesvirus (KSHV) is a common virus with severe outcome and no effective antiviral treatment. KSHV encodes the constitutive active chemokine receptor ORF74 with broad-spectrum CXC-chemokine binding. Here, we leverage ORF74’s mimic of endogenous receptors to design chemokine-based immunotoxins for selective killing of KSHV-infected cells.

**Experimental approach:**

Four CXC-chemokines with high affinity to ORF74 were fused to domain II, IB, and III of *Pseudomonas* exotoxin A to generate fusion toxin proteins (FTPs). FTP-induced cell killing was tested in cells expressing ORF74 or one of four chemokine receptors (CXCR1–4). Internalization of all receptors was probed using SNAP-tagged receptors. Second-generation FTPs were designed from receptor structures and molecular modelling to increase selectivity for ORF74 over CXCR1–4. Finally, antiviral activity of FTPs was tested using genetically engineered KSHV.

**Key results:**

FTPs, based on the agonists (CXCL1, and −8) and inverse agonists (CXCL10 and −12) of ORF74 potently killed ORF74-expressing cells. The inverse agonist based FTPs leveraged constitutive internalization for efficient toxin delivery via ORF74, whereas agonists increased internalization further. CXCL10-FTP had the strongest cell-killing and, as the only FTP, selectivity for ORF74 over its endogenous receptor, CXCR3. Second-generation FTPs improved this selectivity from 25-fold to 126-fold by the mutation (R8D) in CXCL10-FTP, designed to lose ionic interaction within CXCR3’s main binding pocket. Both inverse agonist-based FTPs effectively prevented KSHV-reactivation.

**Conclusion and implications:**

Our findings highlight the versatility of FTPs in precise delivery of toxin payloads and provide a foundation for potential applications in antiviral and anticancer therapies targeting KSHV-associated diseases.

## Highlights

• Kaposi’s sarcoma-associated herpesvirus (KSHV) is widespread and oncogenic.
• Infections with KSHV and related diseases lack effective treatment.
• KSHV encodes the constitutive active receptor ORF74, binding many CXC-chemokines.
• Endogenous chemokines hijacking inspired the fusion toxin proteins (FTPs) design.
• Our FTPs target ORF74, allowing selective killing of KSHV-infected cells.

## Graphical Abstract

**Figure:**
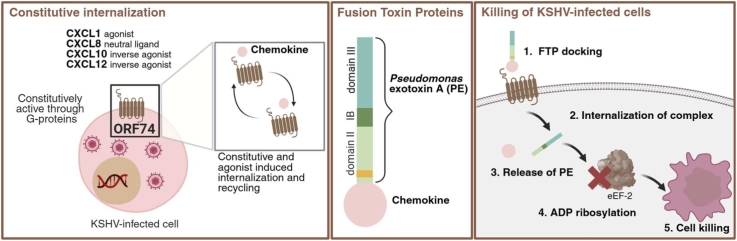

## 1 Introduction

Kaposi's sarcoma-associated herpesvirus (KSHV), also referred to as human herpesvirus 8 (HHV8), belongs to the gammaherpesvirinae and is closely related to Epstein-Barr virus (EBV, HHV4). Like other herpesviruses, KSHV is a large double-stranded DNA virus capable of establishing lifelong latent infections, where reactivation to lytic infection often occurs when the host’s immune system is compromised. This, in turn, can lead to severe morbidity and mortality [1]. KSHV was discovered in 1994 [2] and is associated with two rare lymphoproliferative disorders: primary effusion lymphoma and multicentric Castleman disease [2]. It also has a causative role in Kaposi's sarcoma (KS) [3], a cancer of the skin and mucous membrane, characterized by red skin patches and oral lesions, with around 40,000 yearly cases worldwide and the highest incidence among HIV-infected individuals [4], [5]. Three decades after its discovery, there is still no effective antiviral treatment of KSHV-related diseases [6].

A common feature among herpesviruses is their acquisition of genes involved in immune control, such as components of the chemokine system (chemokine receptors, chemokines, and chemokine binding proteins) [7]. This phenomenon, often referred to as molecular piracy, benefits the viral life cycle by enabling escape from immune surveillance, facilitating virus spreading, and reprogramming host cells to support viral survival [8], [9]. KSHV encodes a chemokine receptor in open reading frame-74, hence denoted ORF74. Like the human chemokine receptors, ORF74 is a G protein-coupled receptor (GPCR) coupling to the G_i_ and G_q_ subunit [10], [11]. However, compared to the human chemokine receptors, ORF74 exhibits high constitutive activity, primarily via the G_q_ subunit [12], [13]. The activity of ORF74 is modulated by a broad range of endogenous chemokines belonging to the CXC-chemokine subfamily: CXCL10 and CXCL12 act as inverse agonists, CXCL8 is a low potent agonist, and CXCL1–3 are full agonists [10], [14], [15], [16]. Thus, ORF74 binds a broad spectrum of chemokines shared with CXCR1 (CXCL6 and CXCL8), CXCR2 (CXCL1–3, CXCL6, and CXCL8), CXCR3 (CXCL10), and CXCR4 (CXCL12), which, besides their role in controlling leucocyte migration during immune homeostasis and inflammation [17], are all involved in the endogenous regulation of angiogenesis [17], [18]. Accordingly, ORF74 has been identified as a potential oncogene in KSHV-related cancers [14], [19], and the constitutive as well as the ligand-induced activity contribute to its oncogenic potential [5], [20].

Viral GPCRs (vGPCR) are not unique to KSHV. The closely related EBV encodes BILF1, an orphan receptor with constitutive activity linked to the virus' oncogenic potential [21]. Similarly, human cytomegalovirus (HCMV) encodes four vGPCRs; the most extensively studied of these is US28, which functions as a broad-spectrum chemokine receptor with high constitutive activity and fast internalization [22], [23], [24]. Like other herpesviruses, HCMV causes a lifelong infection that is usually asymptomatic but can be deadly in immunosuppressed individuals [25]. US28 has emerged as an attractive target for novel therapeutics, including small molecules and single-domain antibodies [26]. Recently, immunotoxins, created as fusion toxin proteins (FTPs) between a selectivity-optimized US28-targeting chemokine moiety and a toxin payload consisting of domain II, IB and III from *Pseudomonas* exotoxin A (PE), demonstrated superiority over conventional nucleoside-based treatment (ganciclovir) [27]. Importantly, this FTP concept proved efficient in killing latently infected monocytes [28] and resulted in > 75 % reduction in the latent HCMV-pool during *ex vivo* treatment of human lungs [29].

**Fig. 1:**
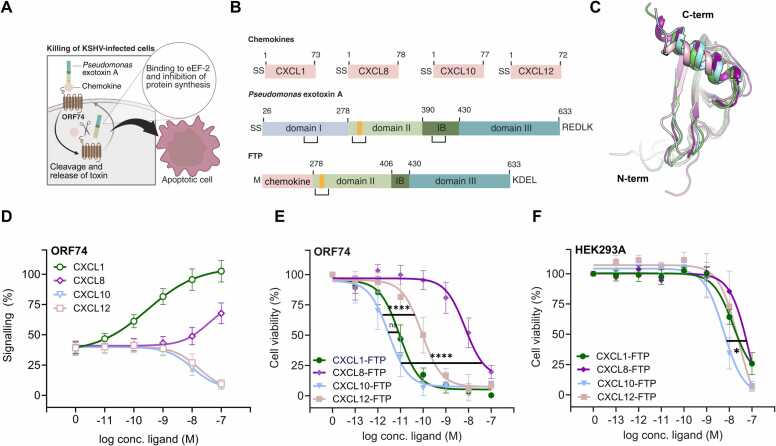
Concept, design and killing by fusion toxin proteins. A Schematic illustration of the proposed mechanism of FTP action. The FTPs bind to ORF74 through their chemokine domain and are internalized via receptor-mediated endocytosis. Following enzymatic cleavage by furin and reduction of the disulfide bond in the endosomes, the toxin moiety undergoes retrograde transport to the endoplasmic reticulum (ER) and is subsequently released into the cytosol, where it ADP-ribosylates elongation factor 2 (eEF-2), leading to inhibition of protein synthesis and ultimately cell death [31]. B Diagram of the FTPs: Top: the chemokines CXCL1, CXCL8, CXCL10, and CXCL12. Middle: Model of *Pseudomonas aeruginosa* Exotoxin A *(*PE): Signal sequence (SS), CD91 receptor binding domain (I), furin cleavage site (orange), cytosol translocation domain (II), domain (IB), and the cytotoxic domain (III). Bottom: a chemokine fused to a modified PE, comprising domain II with the furin cleavage site, a shorter and modified IB domain without the native disulfide bridge, and domain III with an optimized KDEL sequence [32], [33]. C Structural superimposition of CXCL1 (PDB: 1MGS, green), CXCL8 (PDB: 1IL8, purple), CXCL10 (PDB: 1O80, blue), and CXCL12 (PDB: 2J7Z, pink), sequence alignment shown in Supplementary Fig. 1. D Chemokine-induced G_q_-signaling measured as Inositol triphosphate (IP3) accumulation in tetracycline-inducible HEK293 cells expressing ORF74 (125 ng/mL tetracycline). Responses are normalized (%) to the maximum response achieved by CXCL1 (at 100 nM). Data represents means ± SEM of *n* = 3 individual experiments, each performed in duplicates. Tetracycline titration data are shown in Supplementary Fig. 1b. E Cell-killing of tetracycline-inducible HEK293 cells expressing ORF74 (125 ng/mL tetracycline) treated with the indicated FTPs. F Cell-killing of control HEK293A cells treated with the indicated FTPs. Data (E and F) are normalized to cycloheximide (100 %) and buffer (0 %) cell-killing, respectively, and represent means ± SEM of *n* = 4 individual experiments performed in triplicates. logEC_50_ values (listed in Supplementary Table 2) were analyzed using one-way ANOVA with multiple comparisons to the most potent FTP (CXCL10-FTP); P value: 0.0332 (*), 0.0021 (**), 0.00021 (***), < 0.0001 (****), ns = not significant.

The similarities between vGPCRs suggest potential cross-applicability in therapeutic strategies. Exploiting the broad-spectrum chemokine-binding profile [10], [14], [15], [16] and previously established internalization of ORF74 from KSHV [30], the present study describes the development of chemokine-based FTPs that target KSHV-infected cells by interaction with and internalization via ORF74 (Fig. 1A). We designed a series of FTPs by combining a chemokine moiety that targets ORF74 and a toxin payload that kills ORF74 expressing cells. By evaluating the FTP-mediated killing of cells expressing ORF74 and the endogenous chemokines receptors (CXCR1–4) [13], [16], along with an in-depth analysis of the receptors’ internalization patterns, we generated a second generation of FTPs with improved selectivity towards ORF74 over the endogenous receptors. These FTPs offer promising prospects for effectively targeting KSHV-infected cells expressing ORF74 in the future.

## 2 Results

### 2.1 Novel fusion toxin proteins targeting ORF74

To assess whether FTPs could facilitate targeted cell-killing of ORF74-expressing cells, we designed a series of FTPs by fusing chemokines to the translocation and cytotoxic domains (domain II, IB, and III, respectively) of PE (Fig. 1B). The chemokine moieties of the FTPs were chosen based on their high affinity to ORF74 and included an agonist (CXCL1), a low potent agonist (CXCL8), and two inverse agonists (CXCL10 and CXCL12), thus covering a broad activity spectrum [13], [16]. Due to the high structural similarity among chemokines, these FTPs can leverage small sequence variations to achieve selective binding to ORF74 (Fig. 1C and Supplementary Fig. 1a). The concept involves 1) interaction of the FTPs with ORF74 through the chemokine domain; 2) intracellular delivery of the FTP through receptor internalization; 3) release of PE via enzymatic cleavage of the FTP by endogenous furin and reduction of the disulfide bridge in the PE translocation domain 4) retrograde transport of PE to Endoplasmic Reticulum(ER) followed by release to the cytosol 5) ADP-ribosylation of elongation factor 2 (eEF-2), resulting in inhibition of protein synthesis and ultimately cell death (Fig. 1A) [31]. To facilitate these later steps, the native C-terminal sequence (RDELK) was replaced with KDEL, the most common ER retention signal, which binds the KDEL receptor to mediate transport from the Golgi to the ER. This modification has previously been shown to enhance ER localization and overall cytotoxic potency [32], [33].

To evaluate the ability of the FTPs to kill ORF74-expressing cells, we established a tetracycline-inducible HEK293 cell line expressing ORF74. This approach ensured uniform and tunable receptor expression for the *in vitro* killing assay and eliminated the risk of cells not expressing the target as with transient transfection [34]. Consistent with previous studies in COS-7 cells [10], [15], [16], the ORF74-expressing HEK293 cells confirmed CXCL1 to be the most potent agonist, CXCL8 to be a low potent agonist, and CXCL10 and CXCL12 to be inverse agonists in G_q_-signaling, measured as inositol trisphosphate (IP3) accumulation (Fig. 1D and Supplementary Fig. 1b). Likewise, the high constitutive signaling activity of ORF74 was evident in these cells, as judged from increasing tetracycline concentration (Supplementary Fig. 1c). For receptor induction ranging from 125 to 500 ng/mL tetracycline, ligand potencies and basal receptor activity were maintained (Supplementary Fig. 1d, e and Supplementary Table 1), providing a robust foundation for subsequent cell-killing studies with the designed FTPs.

In the killing assay, cells were exposed to the FTPs for 20 hours. Cell viability was then assessed using a cell-impermeable viability reagent, alamarBlue™, which emits red and fluorescent light upon entering living cells. The agonist-based CXCL1-FTP displayed high potency in the picomolar range in killing ORF74-expressing cells (Fig. 1E). Moreover, CXCL1-FTP retained binding to ORF74 and functioned as an agonist, like CXCL1 (Supplementary Fig. 2). In line with its weaker signaling properties, the CXCL8-based FTP exhibited lower killing potency via ORF74 (Fig. 1E and Supplementary Table 2). Interestingly, the FTPs based on the inverse agonists (CXCL10 and CXCL12) could also kill ORF74-expressing cells. While CXCL12-FTP showed intermediate cell-killing potency, CXCL10-FTP emerged as the most potent FTP, being significantly more potent than CXCL8-FTP and CXCL12-FTP and displaying similar high potency as the agonist-based CXCL1-FTP (Fig. 1E and Supplementary Table 2). Unspecific killing in untransfected HEK293A cells was weak with potencies > 1500-fold lower for CXCL1-FTP and CXCL10-FTP, > 300-fold lower for CXCL12-FTP and > 12-fold lower for CXCL8-FTP (Fig. 1F and Supplementary Table 2). As expected, the chemokines alone did not induce any cell-killing (Supplementary Fig. 3).

In summary, two fusion proteins, CXCL1-FTP and CXCL10-FTP, killed cells with high potency. This demonstrates the efficiency of FTPs in delivering toxin payloads into ORF74-expressing cells, facilitating targeted cell-killing.

### 2.2 Basal and ligand-induced internalization of ORF74

**Fig. 2:**
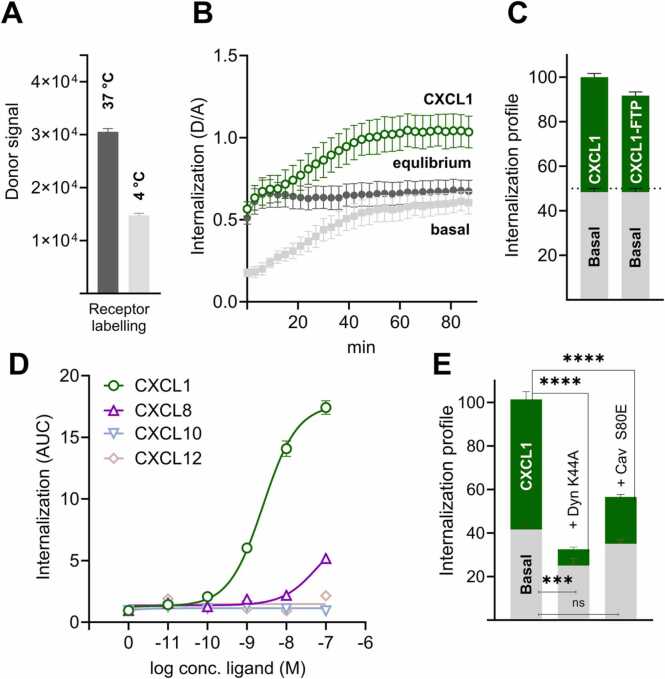
Internalization of ORF74. A Cell surface expression levels of SNAP-tagged ORF74 measured as donor signal. Donor labelling was performed at 37 °C and 4 °C and background corrected (mock transfection). B Real-time internalization, measured as the ratio between donor and acceptor (D/A) emission. Constitutive internalization, measured by receptor labelling at 4 °C, and CXCL1 (100 nM) induced internalization measured by labelling at 37 °C. The internalization (D/A) was measured every 3 min for 90 min. C Internalization profile, shown as normalized area under the curve (AUC) of constitutive (basal) and ligand-induced (100 nM CXCL1 and CXCL1-FTP) internalization in % of total internalization. D Internalization in response to increasing concentration of the indicated chemokines (labelling at 37 °C). Data are reported as AUC from D/A ratio (6–90 min). E Internalization profile of SNAP-tagged ORF74 co-transfected with a dominant negative variant of dynamin (Dyn K44A) or caveolin (Cav S80E). Data reported as (%) normalized area under the curve (AUC) of constitutive internalization and CXCL1 (100 nM) induced internalization of the total internalization achieved with co-transfection with empty vector (first bar). Data represent means ± SEM of *n* = 4–5 individual experiments performed in triplicates. Internalization profiles were analyzed using one-way ANOVA with multiple comparisons to ORF74 (co-transfection with empty vector).

Agonist-induced internalization of ORF74 has been demonstrated previously [30], and constitutive internalization has also been proposed [35] for ORF74 and other vGPCRs [24], [36], [37]. Given the potent killing by both the agonist- and the inverse agonist-based FTPs (CXCL1-FTP and CXCL10-FTP, respectively), we next sought to determine the basal internalization of ORF74 and assess how the different chemokines used in the FTPs influence receptor internalization. This was investigated using a time-resolved diffusion-enhanced resonance energy transfer (DERET) assay using an N-terminally SNAP-tagged ORF74 receptor that enables covalent donor labeling [38]. In the presence of a cell-impermeable acceptor, the DERET donor/acceptor ratio (D/A) describes the time course of receptor internalization [39]. Two different receptor labeling conditions were applied to distinguish basal and ligand-induced internalization. At 4 °C, the low temperature impairs internalization and recycling and only receptors at the cell surface are labeled. This approach allows for determining basal (constitutive) internalization when the temperature gradually increases. In contrast, labeling at 37 °C allows continuous receptor internalization and recycling. At this temperature, an equilibrium is reached between labeled receptors on the cell surface and those inside the cell, resulting in more receptors being labeled at 37 °C compared to 4 °C (Fig. 2A). The constitutive internalization reached a plateau after 50 min, indicating equilibrium between receptor internalization and recycling (Fig. 2B). Subsequent addition of the agonist CXCL1 doubled the internalization at 37 °C (Fig. 2B, C). The internalization profile of CXCL1-FTP was like CXCL1, showing that the C-terminal toxin domains did not prevent internalization (Fig. 2C). CXCL8 only slightly increased internalization at the highest concentration (100 nM) (Fig. 2D), aligning with its low potency in G_q_ signaling. Despite their negative intrinsic activity in G_q_ signaling (Fig. 1C), the two inverse agonists, CXCL10 and CXCL12, did not affect the internalization (Fig. 2D). The presence of the SNAP-tag did not disrupt the basic properties of ORF74 as the constitutive and agonist-induced G_q_ signaling was maintained, and the cell-killing was comparable for ORF74- and SNAP-ORF74-expressing cells, judged by CXCL10-FTP addition (Supplementary Fig. 4).

To further investigate the internalization mechanism, we co-transfected with nonfunctional variants of two key components of endocytosis: the dominant-negative variants of dynamin (Dyn K44A) and caveolin (Cav S80E). Caveolin plays a role in the formation of caveolae in the caveolin-mediated pathway, whereas dynamin is responsible for the pinching of vesicles in both the caveolin- and clathrin-mediated pathways, the latter representing another major endocytic route [37] . We found that CXCL1-induced internalization was significantly reduced in the presence of nonfunctional dynamin and caveolin variants. In contrast, basal internalization was only reduced by the nonfunctional dynamin variant (Fig. 2E).

In conclusion, ORF74 internalizes constitutively and by agonists. Inverse agonists, however, neither promote nor impede internalization, suggesting that the cell-killing by CXCL10-FTP and CXCL12-FTP is mainly driven by constitutive receptor internalization. Basal and agonist-induced internalization mechanisms proceed via distinct pathways: basal internalization occurs via a dynamin-dependent route, whereas CXCL1-induced internalization requires both dynamin and caveolin. This difference in receptor internalization and recycling may influence intracellular delivery, hence, the toxicity of FTPs.

### 2.3 Selectivity towards ORF74 over endogenous chemokine receptors

**Fig. 3:**
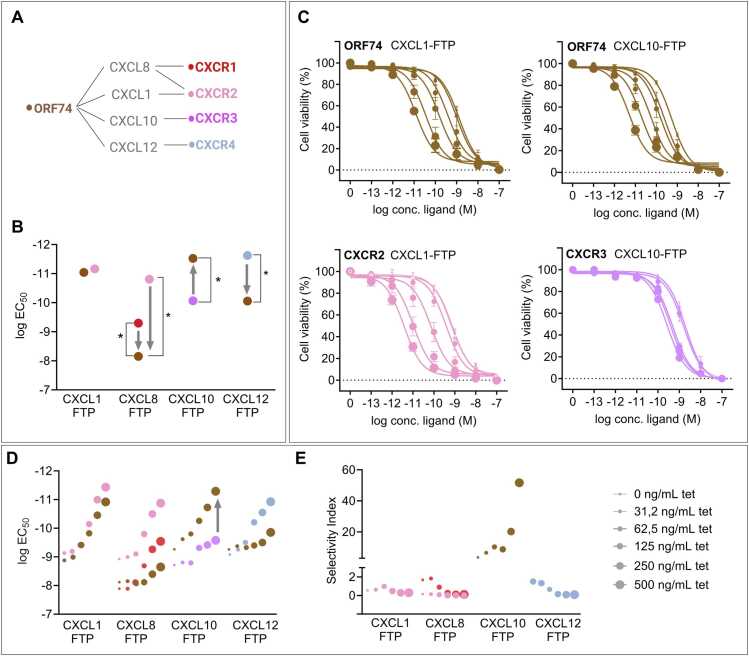
Cell-killing selectivity of FTPs. A Overview of chemokine interactions with ORF74 and endogenous receptors; the color-coding scheme for ORF74 (brown), CXCR1 (red), CXCR2 (pink), CXCR3 (purple) and CXCR4 (blue) applies to all panels. B Cell-killing potency (logEC_50_) of FTPs in tetracycline-inducible HEK293 cells expressing ORF74 (125 ng/mL tetracycline). The higher potency of CXCL10-FTP on ORF74 (brown dots) compared to the endogenous CXCR3 receptor (purple) is indicated with the arrow. LogEC_50_ values are shown in Supplementary Table 2. Data are analyzed using two-way ANOVA with multiple comparison to ORF74, p-value: < 0.001(****). C FTP-mediated cell-killing measured in tetracycline-inducible HEK293 cells expressing the indicated receptor. Increasing tetracycline concentrations (0–500 ng/mL) are illustrated by dot sizes. D Cell-killing potency of FTPs (logEC_50_) from (C) and Supplementary Fig. 6 for increasing tetracycline titrations shown as ORF74 paired with the respective endogenous chemokine receptors. The higher potency of CXCL10-FTP on ORF74 compared to CXCR3 is indicated with an arrow. E Selectivity changes for increasing tetracycline concentrations, calculated as the selectivity index, i.e. fold difference between logEC_50_ for ORF74 and the endogenous receptor (CXCR1, CXCR2, CXCR3, and CXCR4). Data are normalized to cycloheximide (100 %) and buffer (0 %) cell-killing, respectively, and represented as means ± SEM of *n* = 3 individual experiments performed in triplicates.

Having established efficient killing by the FTPs, we went on to determine their selectivity for ORF74 relative to endogenous chemokine receptors. We established tetracycline-inducible HEK293 cell lines of CXCR1, CXCR2, CXCR3, and CXCR4 and used 125 ng/mL tetracycline for receptor induction, as in the previous killing studies. Despite the additional C-terminal bulk of the FTPs, all four chemokine moieties bound to their cognate endogenous receptors, as judged by the potent cell-killing (Fig. 3A, B and Supplementary Table 2). The CXCL1-based FTP showed similar high killing potency in cells expressing CXCR2 and cells expressing ORF74, meaning it was unselective. In contrast to CXCL1, which only binds to CXCR2, CXCL8 is an agonist for CXCR1 and CXCR2. Hence, CXCL8-FTP was tested on both receptors and showed selectivity towards both CXCR1 and CXCR2 over ORF74 (14- and 446-fold higher potency, respectively). Also, the CXCL12-FTP showed higher selectivity (35-fold) towards its endogenous receptor CXCR4. Remarkably, as the only FTP, CXCL10-FTP demonstrated selectivity (25-fold higher potency) for ORF74 over its endogenous receptor CXCR3 (Fig. 3B).

To explore these selectivity patterns, we tested how receptor expression levels affected killing by varying tetracycline concentrations from 31.2 to 500 ng/mL. This allowed us to determine the selectivity index, defined as the fold difference in the killing potency of an FTP between ORF74 and relevant endogenous receptors across different receptor expression levels. The increased receptor expression for increasing tetracycline concentrations was confirmed by binding studies where maximal binding capacity (B_max_) increased with higher tetracycline levels (Supplementary Fig. 5). We observed that the killing potency of CXCL1-FTP increased for both ORF74 and CXCR2 with higher tetracycline concentrations (Fig. 3C and D) with a selectivity index slightly favoring CXCR1 over ORF74 regardless of the receptor expression levels (Fig. 3E). Similar trends were also observed for CXCL8-FTP (acting via CXCR1 and CXCR2), and CXCL12-FTP (via CXCR4) compared to ORF74-expressing cells (Fig. 3D, E and Supplementary Fig. 6). In all cases, a slight increase in selectivity favored the endogenous receptor (Fig. 3E). Interestingly, the inverse agonist-based FTP (CXCL10-FTP) showed a different pattern. Its killing potency correlated positively with ORF74 expression; yet, at CXCR3 (CXCL10’s endogenous receptor), it displayed only a limited window of tetracycline-dependent killing (Fig. 3C and D). Consequently, the favorable selectivity index of CXCL10-FTP for ORF74 over CXCR3 improved from 3- to 60-fold with increasing receptor expression levels (Fig. 3E). Thus, as the only FTP, CXCL10-FTP displayed selectivity towards the viral ORF74 over an endogenous receptor.

### 2.4 CXCR3 shows high constitutive internalization and resembles ORF74

**Fig. 4:**
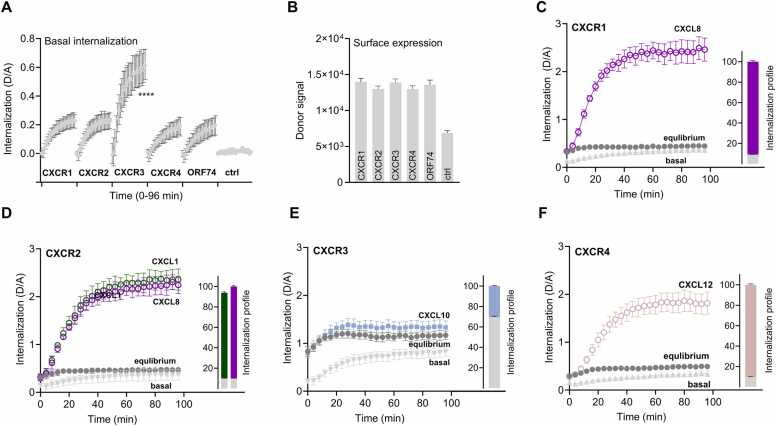
Internalization of chemokine receptors. HEK293A cells transiently transfected with SNAP-tagged CXCR1, CXCR2, CXCR3, CXCR4, ORF74, and empty vector (ctrl). A Constitutive internalization, measured as the ratio between donor and acceptor emissions (D/A), with receptor labelling at 4 °C. Plateau of internalization was obtained by fitting data to a one-phase exponential association model and analyzed with one-way ANOVA using multiple comparisons to ORF74, **** p-value: < 0.001. B Surface expression levels of SNAP-tagged receptor measured as donor signal (labelling at 4 °C) without the presence of the acceptor. C-F Agonist-induced internalization of the indicated receptors measured at equilibrium conditions (receptor labelling at 37 °C). Internalization profile reported as (%) normalized area under the curve (AUC) of constitutive internalization and agonist-induced internalization of the total internalization. The internalization DERET ratio (D/A) is measured every 3 min for 90 min. Data represent means ± SEM of *n* = 5 individual experiments performed in triplicates.

To understand the toxicity profiles of the FTPs via endogenous receptors, we next focused on their internalization. Again, we used the SNAP-tag strategy and measured the constitutive and ligand-induced internalization. Among the four endogenous receptors with ligand overlap with ORF74, CXCR3 stood out with a higher level of constitutive internalization compared to CXCR1, CXCR2, CXCR4, and ORF74 (Fig. 4A). Importantly, receptor expression levels were similar for all receptors (Fig. 4B).

A distinct pattern emerged for the agonist-induced internalization. For CXCR1, more than 90 % of the total internalization was driven by the endogenous agonist (CXCL8) (Fig. 4C). This pattern was also observed for CXCR2 and CXCR4 (Fig. 4D and F), which exhibited minimal basal internalization contribution and high agonist contribution upon addition of CXCL8 and CXCL12, respectively. CXCR3 behaved differently, with less agonist-stimulated internalization (30 % of total internalization) than its substantial basal internalization (Fig. 4E).

In conclusion, the internalization of CXCR1, CXCR2, and CXCR4 was mainly driven by their endogenous agonists, while CXCR3 displayed a high degree of constitutive internalization, like ORF74.

### 2.5 Rational optimization of FTP

**Fig. 5:**
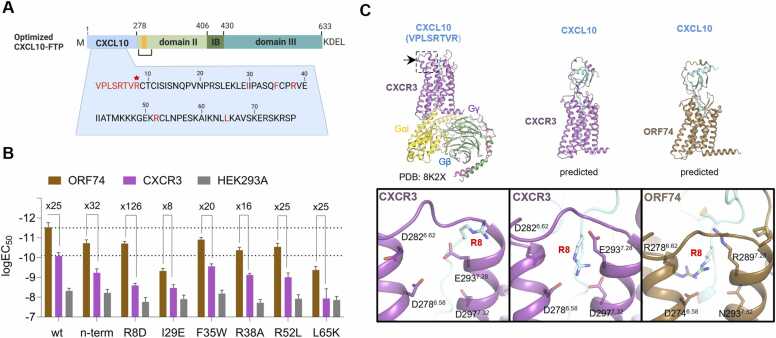
Selectivity optimization of the CXCL10 domain. A Mutations in the CXCL10 chemokine domain of the CXCL10-FTP are indicated in yellow. The star indicates the most selective FTP variant (R8D). B Cell-killing potency (logEC_50_) by the optimized CXCL10-FTPs in stable tetracycline-inducible HEK293 cells expressing ORF74 or CXCR3 (125 ng/mL tetracycline) and in control HEK293A. Raw data represent *n = 4* individual experiments performed in triplicates. Fold differences between logEC_50_ values are indicated in the figure. C Comparison of the cryo-EM structure of CXCL10-CXCR3-Gi (PDB ID: 8K2X, left), predicted models of CXCL10-CXCR3 (center), and CXCL10-ORF74 (right). Only residues 1–8 of CXCL10 are present in 8K2X.pdb, and the region around R8 is zoomed in at the bottom of the figure. CXCL10, CXCR3, and ORF74 are colored blue, purple, and brown, respectively, shown as ribbon structures with key sidechains displayed as sticks. For CXCL10-CXCR3 and CXCL10-ORF74, only the top-ranked predicted structures are shown.

Based on its favorable selectivity for ORF74 over CXCR3 and the low degree of CXCL10-induced internalization via CXCR3, CXCL10-FTP was selected for further optimization. Our main aim was to improve the selectivity by introducing modifications in the chemokine moiety that decreased the affinity for CXCR3. The modifications (Fig. 5A) involved six point-mutations, focusing on changing charge (R8D, I29E, R38A, R52L, and L65K) or introducing steric hindrance (F35W) and the replacement of the CXCL10 N-terminus (VPLSRTV) with that of CXCL12 (KPVSLSY), which does not bind CXCR3 but binds ORF74 [9] (named *n-term)*. In the cell-killing assay, the seven variants generally exhibited reduced potency towards both ORF74 and CXCR3 expressing cells (Fig. 5B). For the variants F35W, R38A, R52, L65K, and *n-term*, the potencies were equally reduced, and the selectivity index remained around 25-fold. In contrast, the I29E variant showed the lowest selectivity with less than 10-fold potency difference. In CXCL10, the arginine at position 8 (R8), placed before the CXC motif, has been recognized as critical for binding to CXCR3 [40], [41]. In agreement with the binding role of R8, the R8D variant exhibited diminished toxicity towards CXCR3-overexpressing cells. Although its toxicity for ORF74 decreased slightly, this FTP demonstrated an improved selectivity index (126-fold) due to a larger potency loss for CXCR3 (Fig. 5B).

The cryo-EM structure of the CXCL10-CXCR3-Gi complex [42], combined with the ColabFold [43] predicted CXCL10-CXCR3/ORF74 models could explain how the CXCL10-FTP with the R8D mutation confers receptor selectivity (Fig. 5C and Supplementary Fig. 7). In the cryo-EM structure, the CXCL10 globular core is extremely flexible on CXCR3, and R8 interacts with E293^7.28^ of CXCR3, where ORF74 encodes R289^7.28^ at the corresponding position (Fig. 5C, superscript denotes Ballesteros-Weinstein numbering for class A GPCRs [44]). The R8D mutation would lead to a loss of ionic interaction and gain of electrostatic repulsion with E293^7.28^ of CXCR3. At the same time, it would cause a loss of pi-pi stacking and gain of an ionic interaction with R289^7.28^ of ORF74 (Fig. 5C and Supplementary Fig. 8), explaining the dramatic and moderate attenuation in the killing potency via CXCR3 and ORF74, respectively. In the predicted CXCL10-CXCR3 structures, CXCL10 docked into the chemokine-binding groove more deeply, and R8 fits into the pocket formed by two layers of acidic residues, D278^6.58^/D297^7.32^ and D282^6.62^/E293^7.28^ of CXCR3. ORF74's pocket is more basic and comprises D274^6.58^/N293 ^7.32^ and R278^6.62^/R289^7.28^, also confirmed in the recently resolved ORF74 structure [45]. This drives the improved ORF74 selectivity by the R8D mutation.

In summary, the R8D substitution in the CXCL10-FTP, although reducing potency against both ORF74 and CXCR3, significantly improved selectivity, showing that modification of the chemokine moiety is a viable strategy for fine-tuning FTP selectivity for ORF74 over CXCR3.

### 2.6 Viral Reactivation

**Fig. 6:**
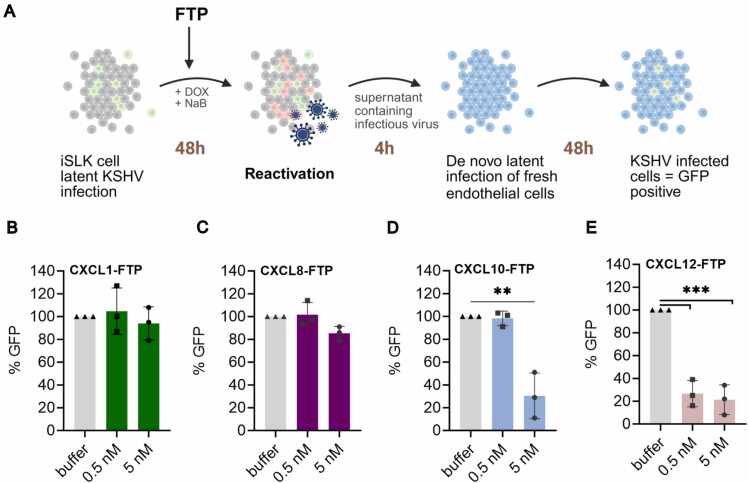
Effect of FTPs on KSHV latently infected cells. A KSHV-infected iSLK cells in latent state, the viral genome has green fluorescent protein (GFP) constitutively expressed and red fluorescent protein (RFP) under a lytic promoter. The cells were induced with doxycycline (DOX) and sodium butyrate (NaB) to undergo lytic reactivation, in the presence of buffer, 5 nM or 0.5 nM FTP. Supernatants, including titers, were isolated and used to infect fresh Tert-immortalized microvascular endothelial (TIME) cells for 4 h. 48 h post titer-infection, GFP was quantified as a proxy for infectious virus. B-E Data are normalized to the GFP signal of TIME cells with vehicle treatment (100 % reactivation). Data represent means ± SEM of n = 5 experiments performed in duplicates. Data was analyzed with an unpaired *t*-test comparing control to FTP conditions, P-value: 0.0021 (**), 0.00021 (***).

To evaluate the effect of FTPs in virus-infected cells, we assessed viral reactivation from latent infection using an inducible iSLK-KSHV cell line carrying a selectable green fluorescent protein (GFP) expressing KSHV genome together with a red fluorescent protein (RFP) under a lytic promotor [46]. Reactivation was induced using doxycycline and sodium butyrate while exposing the cells to FTPs at 5 nM and 0.5 nM for 48 h. Supernatants were then collected and used to infect fresh endothelial cells for four hours. The GFP expression was measured 48 h post-infection and served as an indicator of infectious virus production during FTP treatment (Fig. 6A). Exposure to CXCL1-FTP and CXCL8-FTP did not alter virus production compared to buffer-treated control cells (Fig. 6B and C). In contrast, CXCL10-FTP exhibited a 70 % reduction in infectious virus production at 5 nM. Similarly, CXCL12-FTP reduced viral reactivation by 79 % and 76 % at 5 nM and 0.5 nM, respectively (Fig. 6D, E). Control experiment confirmed that the FTP did not affect endothelia cell viability, indicating that the observed reduction reflects reduced reactivation of iSLK cells rather than direct toxicity to target cells (data not shown).

The finding that only the inverse agonist-based FTPs, CXCL10- and CXCL12-FTP reduce viral reactivation suggests that antiviral effect depends on the pharmacological profile of the chemokine moiety. This is consistent with our internalization data, showing that agonist-induced and constitutive internalization follow distinct pathways, likely impacting FTP delivery and antiviral activity. These results highlight the potential of inverse agonist-FTPs for targeted control of KSHV infection.

## 3 Discussion and Conclusions

Most antiviral treatments target the viral replication machinery, such as inhibitors of viral replication and inhibitors of virus particle maturation. Thus, more than 95 % of all antiviral therapies target enzymes, whereas only very few drugs are directed against viral membrane-expressed proteins [47]. Among the few approved drugs for membrane-expressed targets are amantadine and rimantadine, targeting the viroporin M2 from Influenza A virus, and palivizumab and nirsevimab, targeting the F protein in human respiratory syncytial virus [48], [49]. Less than half of all human herpesviruses are treated with DNA polymerase inhibitors, as herpes simplex I and II, varicella zoster and HCMV infections are effectively treated this way [50]. For KSHV, the current treatment also relies on DNA polymerase inhibitors. However, these show limited efficiency [6]. Therefore, there is a general need for new, effective antiviral treatments.

Immunotoxins targeting viral GPCRs, such as ORF74, represent a complementary and potentially more selective strategy. By directing the toxin to virus-encoded receptors that are uniquely expressed on infected cells, these agents achieve high specificity and thereby minimize off-target toxicity. Because their mode of action relies on cell killing rather than replication inhibition, they may also overcome conventional resistance mechanisms. Chemokine-based targeting further enhances precision by mimicking natural ligand–receptor interactions, as shown for the CMV-encoded receptor US28 [28], [29], [51].

We aimed to develop FTPs targeting the KSHV-encoded receptor ORF74 with high efficacy and selectivity. The first series was based on endogenous chemokines with known profiles at ORF74 as either agonists or inverse agonists [16]. With these, we successfully targeted ORF74-expressing cells. Notably, the FTP based on the inverse agonist CXCL10 showed unexpectedly high potency, likely driven by basal internalization.

Successful delivery of FTPs relies on two essential components: sufficient binding of the FTP to the target cell surface protein and internalization of the target. In previous studies targeting the HCMV-encoded receptor US28 with FTPs [27], [52], the chemokine moiety was based on the CX3CL1, which has a unique membrane-bound structure in its full-length form. This includes an N-terminal chemokine domain, a mucin-like stalk, and a transmembrane helix in the C-terminal [53]. Consequently, the chemokine domain in CX3CL1 is inherently associated with a C-terminal extension. In contrast, the CXC-chemokines in the FTPs designed in the present study exclusively consist of a chemokine domain with no inherent extensions. Fusing part of *Pseudomonas* exotoxin A (344 amino acids) to the CXC-chemokine domain (73–77 amino acids) substantially increases size and structural complexity. Despite this, the receptor binding remained intact, highlighting the versatility and flexibility of the chemokine system for intracellular delivery of fusion toxin proteins.

The present study shows high basal (constitutive) internalization of ORF74, which aligns with previous data [30], [35]. Moreover, CXCL1 induced internalization of ORF74, consistent with its agonism in G protein signaling [14], [15], [16]. Intriguingly, the inverse agonists, CXCL10 and CXCL12, did not affect internalization, deviating from observations with other inverse agonists and/or antagonists of GPCRs, like GIP(3−30)NH_2_ and AMD11070, which effectively prevents GIPR and CXCR4 internalization, respectively [54], [55]. This implies that the intracellular delivery of the inverse agonist-based FTPs (CXCL10-FTP and CXCL12-FTP) is primarily driven by basal internalization and that this is an efficient way to drive cell-killing. Our findings for dominant negative forms of dynamin and caveolin support different internalization routes for agonists (that induce internalization) and inverse agonists (that “follow” the basal internalization) as part of the mechanism of toxicity for the FTPs. Similar observations have been reported for other receptors, such as the M_3_-receptor, CCR2, and GPR183, where the constitutive and ligand-induced internalization utilize different endocytosis pathways [38], [56].

In our effort to enhance selective toxicity against ORF74-expressing cells, we pursued a rational design of the CXCL10 moiety. The increased selectivity of the modified FTP-CXCL10 R8D towards ORF74 can be explained by electrostatic repulsion in the CXCR3 binding pocket and only moderate loss of potency for ORF74. However, further refinement is necessary to reduce off-target side effects for future targeting of ORF74 to kill KSHV-infected cells, preferentially aiming for a similar high selectivity (>1000-fold) obtained for the US28-targeting FTPs over the endogenous CX3CR1 [52]. Given the substantial basal internalization of CXCR3, two strategies can be pursued in future studies to decrease FTP toxicity through CXCR3: 1) further *reduction* of binding of CXCL10 to CXCR3, *i.e.,* lower affinity; or 2) modifying CXCL10 to become an *antagonist* for CXCR3, which would prevent agonist-induced internalization, and perhaps even retain the receptor at the cell surface as observed for other GPCRs [54], [55].

We showed that the inverse agonist (CXCL10 and CXCL12) based FTPs could reverse the viral reactivation by 70–79 %, while no effect was seen for the agonist-based (CXCL1 and CXCL8) FTPs. This is consistent with reactivation studies of murine gammaherpesvirus 68 (MHV68), the murine homolog of KSHV that also encodes a CXC-chemokine receptor in open reading frame 74 (ORF74). Previous data have shown that CXC-chemokine antagonists (or inverse agonists) inhibit virus reactivation from latency, while agonists promote this transition through their targeting of MHV68-ORF74 [57]. Based on our data, we anticipate similar behavior of the chemokines for KSHV-ORF74, which would offer a dual advantage to FTPs based on inverse agonists—selectively killing ORF74-expressing cells while limiting reactivation from latency. CXCR4 is widely expressed and present in endothelial cells [58], [59] and has been identified as endogenously expressed in HEK293 cell lines [60], [61], [62], which could explain the higher toxicity of the CXCL12-FTP in the endothelial cell line.

In conclusion, our studies suggest that targeting ORF74 could be a promising strategy for treating KSHV-associated cancers*. In vivo* studies are needed to assess this approach fully. Given the challenges of KSHV propagation in nonhuman mammals, murine models of MHV68 infection expressing ORF74-MHV68 offer a valuable alternative tool for further investigation [9]. The FTP approach has not been previously proven for oncogenic viruses, where cell killing is an obvious strategy going forward. Hence, it could be extended to target the closely related BILF1 receptor in EBV. Moreover, targeting endogenous GPCRs like CXCR4, implicated in various cancers would broaden its therapeutic potential across multiple cancer types.

## 4 Materials and methods

### 4.1 Receptor constructs

For transient transfections ORF74 and SNAP-ORF74, -CXCR1, -CXCR2, -CXCR3, and -CXCR4 were cloned into pcDNA 3.1 vector. For the SNAP-tagged receptors, the receptor sequence was proceeded by an N-terminal IL2R1-SS signal sequence, followed by the FLAG and SNAP coding sequences.

For stable transfection, CXCR1, CXCR2, CXCR3, CXCR4, and KSHV-ORF74 were cloned into the pcDNA5/FRT/TO vector and synthesized by Genscript. The flp recombinase vector (pOG44) was purchased from Thermo Fisher Scientific (cat no. R78007) together with the HEK293 Flp-in™ –T-REx cell Line.

### 4.2 Cell culturing and generation of stable inducible cell lines

HEK293A (RRID: CVCL_6910) were cultured at 5 % CO_2_ and 37 °C, 95 % relative humidity (RH) in Dulbecco’s modified Eagle’s medium (DMEM)-GlutaMAX (Gibco) supplemented with 10 % fetal bovine serum (Gibco), 180 units/mL penicillin, and 45 µg/mL streptomycin (complete medium). COS-7 cells (RRID: CVCL_0224) were cultured in the same conditions described above but supplemented with 2 mM glutamine (Gibco). The cell lines were applied in further experiments between passages 10 and 30.

The tetracycline-inducible cell lines, HEK293 Flp-In™ T-REx™ (Thermo Fischer Scientific, cat. no R78007) was cultured in complete media with 15 µg/µL blasticidin (Gibco) and 100 µg/µL Zeocin (Gibco). Lipofectamine 2000 (Thermo Fisher Scientific) (36 µL) was used for transfection. In short, the HEK293 Flp-In™ T-REx™ cells (passage 5–10) were seeded at a density of 1.5 × 10^6^ cells per T25 flask 24 h before transfection. The next day, the transfection mix was prepared as follows: 1.5 µg of pcDNA5/FRT/TO vector containing the gene coding for a receptor of interest (ORF74, CXCR1, CXCR2, CXCR3, or CXCR4) was mixed with 15 µg of pOG44 allowing integration of the receptor into the host genome, in 450 µL Opti-MEM (Thermo Fisher Scientific). Prior to adding the transfection mix to the cells, the media was changed to complete media with blasticidin 15 µg/µL only. The transfection was terminated the following day by changing the media/transfection mix to complete media supplemented with blasticidin 15 µg/µL. Two days after transfection, selection media, composed of the complete media supplemented with 15 µg/µL blasticidin and 130 µg/µL hygromycin B (Gibco). After 2–4 weeks successful clones appeared and were pooled. The obtained tetracycline-inducible cell lines were further applied in experiments between passage 10 and 30.

### 4.3 Antiviral Fusion-Toxin Proteins (FTPs)

The FTP DNA constructs were cloned into the pET21a(+) vector (Novagen) at GenScript. The expression vector was transformed into *E. coli* BL21-Gold (DE3) cells (Agilent Technologies) by heat shock transformation following the manufacturer’s protocol. Protein expression was induced with 0.5 mM IPTG for 3 h at 37 °C followed by inclusion body isolation. Cell pellets were resuspended in lysis buffer (50 mM Tris-HCl, 100 mM NaCl, 5 mM EDTA-NaOH, 1 mM Benzamidine, 3 mM DTT, 1 mM PMSF, 10 mM MgCl_2_ 6 H_2_O and 10 g/L DNAse I pH 8.0) and lysed by sonication. To purify the inclusion bodies, the cell pellet was resuspended and sonicated two times in wash buffer (50 mM Tris-HCl, 300 mM NaCl, 0.25 % (w/v) Na-deoxycholate, and 3 mM DTT, at pH 8.0) and a third time in wash buffer without Na-deoxycholate. To solubilize the inclusion bodies, the pellet was dissolved in 3 M Guanidine-HCl, 100 mM Tris-HCl, 5 mM EDTA-NaOH, and 5 mM DTT, at pH 8.0, followed by incubation at room temperature for 30–60 min and centrifugation (15 000 xg) to remove insoluble material. The solubilized and denatured inclusion bodies were dialyzed against 1x PBS at 4 °C for 30 min, with overnight subsequent refolding in PBS containing 0.2 mM cystine and 1 mM cysteine at pH 7.2. To remove potential precipitate, the dialyzed sample was centrifuged (15 000 xg) and further supplemented with three volumes of 50 mM HEPES-NaOH and 5 mM MgCl_2_ 6 H_2_0 pH 7.2 at room temperature and filtered through a 0.2 µm filter (Merck).

For purification, Fast Protein Liquid Chromatography (FPLC) ÄKTA Purifier system (GE Healthcare) was used with HiScale 16/20 column (GE Healthcare) packed with AEX resin (SOURCE 30Q). The protein was bound to the column and eluted with a linear salt gradient by increasing the high salt buffer (50 mM HEPES-NaOH, 1 M NaCl, and 5 mM MgCl_2_ 6 H_2_0 pH 7.2) from 0 % to 30 % over six column volumes. The fractions with the protein of interest were concentrated in Amicon ultrafilter with a 50 kDa MWCO centrifugal device and loaded onto HiLoad 16/600 Superdex 75 pg column (60 cm×16 mm) (Cytvia) equilibrated in PBS at pH ∼7.4. The relevant fractions were pooled and concentrated in an Amicon ultrafilter with a 50 kDa MWCO. The fractions were analyzed by SDS-PAGE throughout the purification process, and the protein was stored at 4 °C until use.

### 4.4 Signaling (Inositol trisphosphate (IP3) accumulation) assay

The IP3 assay was performed with transient transfected COS-7 cells and stable inducible cells. For transient transfections, the calcium phosphate method was used as described before [63].

After 5 h, the transfection was terminated, and 35 000 cells per well were seeded in poly-D-lysine-coated white 96-well plates. For stable tetracycline-inducible cells, 10 000 cells per well were seeded. The following day, Myo-[2-^3^H(N)]-inositol (Revvity) at a concentration of 5 µL/mL was added. For the inducible cell line, receptor expression was induced with tetracycline 125 ng/mL (Sigma-Aldrich) unless otherwise stated. On day three, the cells were washed twice with HBBS buffer (Gibco), followed by the incubation with the assay buffer (HBBS) prewarmed to 37 °C and supplemented with 10 mM LiCl (Sigma-Aldrich). Ligands were added from 10 pM to 100 nM and incubated for 90 min at 37 °C. Next, the plate was placed on ice and wells were aspirated, followed by the extraction of cells with 40 µL 10 mM formic acid (Poch) per well and 30-minute incubation on ice. Cells were lysed with a lysis solution containing 200 mM NaOH and 1 % sodium dodecyl sulfate (SDS, Sigma-Aldrich) and left shaking at room temperature for 30 min. Lastly, radioactivity was determined using a Packard Top Count NXT scintillation counter (PerkinElmer). Determinations were made in duplicates.

### 4.5 Cell-killing assay

Stable tetracycline-inducible cell lines expressing ORF74, CXCR1, CXCR2, CXCR3, or CXCR4- were seeded at 10.000 cells per well in black in poly-D-lysine-coated 96-well tissue culture plates. One day after seeding, the receptor expression was induced with 125 ng/mL tetracycline (unless otherwise stated). The day after induction, the FTP was applied in a concentration range from 10 pM to 100 nM, and alternatively, chemokine buffer (negative control, consisting of 1 mM acetic acid (Poch) and 1 g/L bovine serum albumin (BSA, Sigma-Aldrich) at pH 4.5) or cycloheximide (positive control for cell-killing). The cells were incubated for 12–20 h at 37 °C at 10 % CO_2_. On day four, the cell viability was estimated by incubating with alamarBlue™ Cell Viability Reagent (Invitrogen) for 4 h at 37 °C and 10 % CO_2_. Data was collected using a Synergy HT plate reader (excitation/emission = 540 nm/585 nm). The cell-killing assay was also performed with transient transfected HEK293A cells using similar transfection as described in the inositol trisphosphate (IP3) accumulation assay. Determinations were made in triplicates.

### 4.6 Real-time receptor internalization

The assay is based on diffusion-enhanced resonance energy transfer (DERET) between SNAP-tagged GPCRs labelled with a donor (Tag-lite SNAP-Lumi4-tb) and a cell impermeable acceptors molecule (fluorescein-O′-acetic acid). The donor emission is quenched by the acceptor when the donor is located at the cell membrane. Upon receptor internalization, the quenching is lost, and the donor signals become greater over time. The SNAP-tagged receptor-DNA (5–12 ng per well) and Lipofectamine 2000 (0.075 ng per well) were mixed in a total volume of 50 µL Opti-MEM. An additional 11 ng per well of Dyn K44A, Cav S80E, or empty vector was added for co-transfection studies. After 20 min, the transfection mix was combined with cells (20 000 cells per well) and seeded in poly-D-lysine-coated white 384-well plates. The transfection was terminated after 24 h by changing the media, and the following day, the assay was performed. The SNAP-tagged receptor and mock-transfected cells (empty vector) were labeled with 10 µL of 100 nM Tag-lite SNAP-Lumi4-tb (energy donor, Cisbio cat.no SSNPTBX) for 60 min at 37 °C or 4 °C to study ligand-regulated or constitutive internalization, respectively. The cells were then washed four times with internalization buffer (HBSS supplemented with 1 mM CaCl_2_, 1 mM MgCl_2_, 20 mM HEPES, and 0.1 % BSA pH 7.4), for analyzing the internalization, the cells were treated with 10 µL 100 µM preheated/cold fluorescein-O′-acetic acid (energy acceptor, Sigma-Aldrich cat. no. 88596-5MG-F). A Viaflow 384 well pipette was used to add 10 μL ligand per well for recordings with ligands. Internalization was measured every third minute for 90 min at 37 °C using PerkinElmer EnVision 2014 Multilabel Reader (Excitation/emission 340 nM/ 520 and 610 nm). The area under donor/acceptor ratio curves was used for the dose-response curves as a function of time. SNAP-tagged receptor surface expression was quantified as the donor emission in the absence of fluorescein, and the amount of DNA was adjusted to achieve similar donor signals between receptor types. Determinations were made in triplicates.

### 4.7 Competition binding assay

Stable Inducible HEK293 Flp-in™ T-REx cells expressing ORF74, CXCR1, CXCR2, CXCR3, or CXCR4 were seeded in poly-D-lysine-coated 96-well plates in a cell density between 10 000 – 50 000 cells per well, aiming to obtain 5–10 % specific binding of ^125^I-CXCL8 or ^125^I-CXCL10 (PerkinElmer). The following day, receptor expression was induced with 125 ng/mL tetracycline (unless otherwise stated). On day three, the cells were incubated with 15–40 pM of ^125^I-CXCL8 and increasing competing ligand (10 pM to 100 nM) in binding buffer (50 mM HEPES buffer, 1 mM CaCl_2_, 5 mM MgCl_2_ and 0.5 % BSA, at pH 7.2). After incubation for 3 h at 4 °C, the cells were washed twice in ice-cold binding buffer and lysed using 200 mM NaOH with 1 % SDS for 30 min. The samples were analyzed by the Wallac Wizard 1470 Gamma Counter (PerkinElmer). Determinations were made in duplicates. The detected binding data were used to calculate the total number of receptors available for ligand binding (Bmax). Competitive binding data were normalized to the maximum specific response for each individual experiment. Sigmoid curves were logistically fitted with a Hillslope of 1.0. Bmax was derived from the competitive binding curves using the equation: B_max_ = (B_0_ × IC_50_)/[L], where B_0_ represents the total specific binding, and [L] is the ligand concentration. Kd was calculated using the formula: Kd = IC_50_ – [L] [64].

### 4.8 Structure predictions

All structure predictions were performed using ColabFold [46], a modified version of Google’s AlphaFold2 and AlphaFold2 multimer [33], via Google Colaboratory. The structures of CXCL10 (residues 22–98, 1–77 as mature form) were predicted as complexes with either CXCR3 (residues 1–337) or ORF74 (residues 1–342) with or without the R8D mutation in the chemokine. Five structures were predicted for each complex without providing any template, and the models were relaxed with Amber [65]. The folding of CXCL10, CXCR3, ORF74, and the overall docking modes concerning the chemokine recognition site (CRS)1.5 and CRS3 were predicted with high to moderate quality (Supplementary Fig. 8) For the top-ranked wild-type complexes, PAE plots were generated with PAE viewer with pseudo-crosslinking info only as markers of the contact sites between R8 (CXCL10) and the acidic cluster. Figures showing these models and the cryo-EM structure of CXCL10-CXCR3-Gi (PDB ID: 8K2X) [42] were prepared using PyMol (Schrödinger).

### 4.9 Viral reactivation assay

The lytic replication assay was performed as described before [69] using iSLK cells harboring a BAC16-KSHV viral genome and an integrated, inducible replication and transcription activator (RTA)-expressing locus [66]. The viral genome has green fluorescent protein (GFP) constitutively expressed and red fluorescent protein (RFP) under a lytic promoter. The cells were maintained in DMEM containing 10 % FBS, 1 % penicillin-streptomycin, and 1 % L-glutamine selected with puromycin (10 mg/mL), G418 (95 mg/mL), and hygromycin B (50 mg/mL). The cells were induced to undergo lytic replication using doxycycline and sodium butyrate in the presence or absence of FTPs (5 nM or 0.5 nM). After 48 h of lytic induction, the supernatant from all samples was collected and spun down at 2000 rpm to remove cellular debris. Supernatants were used to infect fresh Tert-immortalized microvascular endothelial TIME cells by incubating cells with cell-free supernatant supplemented with Polybrene (8 μg/mL) in 6- or 12-well plates for 4 h. Since BAC16-KSHV expresses GFP, the GFP levels served as an indicator of lytic reactivation following FTP treatment in iSLK cells. The relative levels of GFP were determined by Typhoon fluorescent imager at 48 h post supernatant infection and analyzed using Image-J software.

### 4.10 Data analysis

Data analysis was performed using GraphPad 9.0 software. LogEC_50_ with SEM was determined by fitting normalized data to a logistic function (sigmoidal response) with a Hill slope of 1 or −1. LogEC_50_ values were analyzed using either one-way or two-way ANOVA by multiple comparison to ORF74 (unless otherwise stated). The p-values are identified as P-value: 0.0332 (*), 0.0021 (**), 0.00021 (***), < 0.0001 (****), ns = not significant. Kinetic parameters for internalization (*e.g.* Plateau) were determined by fitting data using a one-phase association model and analyzed with one-way ANOVA using multiple comparison to ORF74. For the viral reactivation assay, data was analyzed with an unpaired *t*-test comparing control to FTP conditions, P-value: 0.0021 (**), 0.00021 (***).

## Funding

This project has received funding from the European Research Council (ERC) under Horizon Europe (grant agreement no. 101055152 to MMR), the 10.13039/501100009708Novo Nordisk Foundation (NNF20OC0062899 to MMR and NNF18OC0033926 to 10.13039/501100006130BBK), the 10.13039/100012774Innovation Fund Denmark (0153–00238B to DFK, TNK, and MGJ), Hørslev-Fonden (legat 2024 to AKD and MMR), and Japan Society for the Promotion of Science (JSPS) KAKENHI (Grant Number JP24K01965 to NT).

## CRediT authorship contribution statement

**Dagmar Fæster Kildedal:** Writing – original draft, Visualization, Investigation, Formal analysis. **Anna Katarzyna Drzazga:** Writing – review & editing, Investigation, Formal analysis. **Anjali Sharma:** Investigation. **Christian Berg:** Investigation. **Astrid Norup Winther:** Investigation. **Laura Krogh-Hansen:** Investigation. **Martin Gustavsson:** Supervision, Methodology. **Birthe B. Kragelund:** Supervision, Methodology. **Jon Våbensø:** Writing – review & editing. **Michael Lagunoff:** Methodology. **Naotaka Tsutsumi:** Writing – original draft, Investigation, Conceptualization. **Thomas N. Kledal:** Methodology, Funding acquisition, Conceptualization. **Mads G. Jeppesen:** Methodology, Funding acquisition, Conceptualization. **Mette M. Rosenkilde:** Writing – review & editing, Writing – original draft, Methodology, Funding acquisition, Conceptualization.

## Declaration of Competing Interest

The authors declare the following financial interests/personal relationships which may be considered as potential competing interests:

Mette M. Rosenkilde reports financial support provided by European Research Council under Horizon Europe (grant agreement no. 101055152), Novo Nordisk Foundation (NNF20OC0062899), and Hørslev-Fonden (legat 2024).

Birthe B. Kragelund reports financial support provided by Novo Nordisk Foundation (NNF18OC0033926).

Dagmar F. Kildedal reports financial support provided by Innovation Fund Denmark (0153–00238B).

Anna K. Drzazga reports financial support provided by Hørslev-Fonden (legat 2024).

Thomas N. Kledal reports financial support provided by Innovation Fund Denmark (0153–00238B).

Mads G. Jeppesen reports financial support provided by Innovation Fund Denmark (0153–00238B).

Naotaka Tsutsumi reports financial support provided by Japan Society for the Promotion of Science (JSPS) KAKENHI (Grant Number JP24K01965 to NT).

Mette M. Rosenkilde reports a relationship with Synklino A/S that includes: board membership, consulting or advisory, equity or stocks, and funding grants.

Thomas N. Kledal reports a relationship with Synklino A/S that includes: employment, equity or stocks, funding grants, non-financial support, and travel reimbursement.

Mads G. Jeppesen reports a relationship with Synklino AS that includes: employment, equity or stocks, funding grants, non-financial support, and travel reimbursement.

Dagmar F. Kildedal reports a relationship with Synklino AS that includes: employment, funding grants, and non-financial support.

Anna K. Drzazga reports a relationship with Synklino AS that includes: employment, funding grants, non-financial support, and travel reimbursement.

Mette M. Rosenkilde has patent #2010/0048470 A1. Immunotoxins for the treatment of diseases related to CMV infection. issued to Synklino A/S, Copenhagen, Denmark, patent #WO2021/008840 A1. FUSION TOXIN PROTEINS FOR TREATMENT OF DISEASES RELATED TO CMV INFECTIONS issued to Synklino A/S, Copenhagen, Denmark, and patent #WO/2022/184825. COMPOSITIONS FOR EX VIVO ORGAN CARE pending to Synklino A/S, Copenhagen, Denmark.

Mads G. Jeppesen has patent #2010/0048470 A1. Immunotoxins for the treatment of diseases related to CMV infection. issued to Synklino A/S, Copenhagen, Denmark, patent #WO2021/008840 A1. FUSION TOXIN PROTEINS FOR TREATMENT OF DISEASES RELATED TO CMV INFECTIONS issued to Synklino A/S, Copenhagen, Denmark, and patent #WO/2022/184825. COMPOSITIONS FOR EX VIVO ORGAN CARE pending to Synklino A/S, Copenhagen, Denmark.

Thomas N. Kledal has patent #2010/0048470 A1. Immunotoxins for the treatment of diseases related to CMV infection. issued to Synklino A/S, Copenhagen, Denmark, patent #WO2021/008840 A1. FUSION TOXIN PROTEINS FOR TREATMENT OF DISEASES RELATED TO CMV INFECTIONS issued to Synklino A/S, Copenhagen, Denmark, and patent #WO/2022/184825. COMPOSITIONS FOR EX VIVO ORGAN CARE pending to Synklino A/S, Copenhagen, Denmark.

Other authors they declare that they have no known competing financial interests or personal relationships that could have appeared to influence the work reported in this paper.

## Data Availability

Data will be made available on request.
