# Supplementary material for: Targeting of Kaposi’s sarcoma-associated herpesvirus by immunotoxins directed against the viral G protein-coupled receptor, ORF74
Source: Biomed Pharmacother. 2026 Feb;195:118797. doi: 10.1016/j.biopha.2025.118797 (PMC12867767; doi:10.1016/j.biopha.2025.118797)
Supplement: Supplementary file 1 — Supplementary material [file mmc1.pdf]

Supplementary Figures

Targeting of Kaposi's Sarcoma-associated Herpesvirus by Immunotoxins Directed Against the Viral G Protein-Coupled Receptor, ORF74.

Dagmar Fæster Kildedal et al.

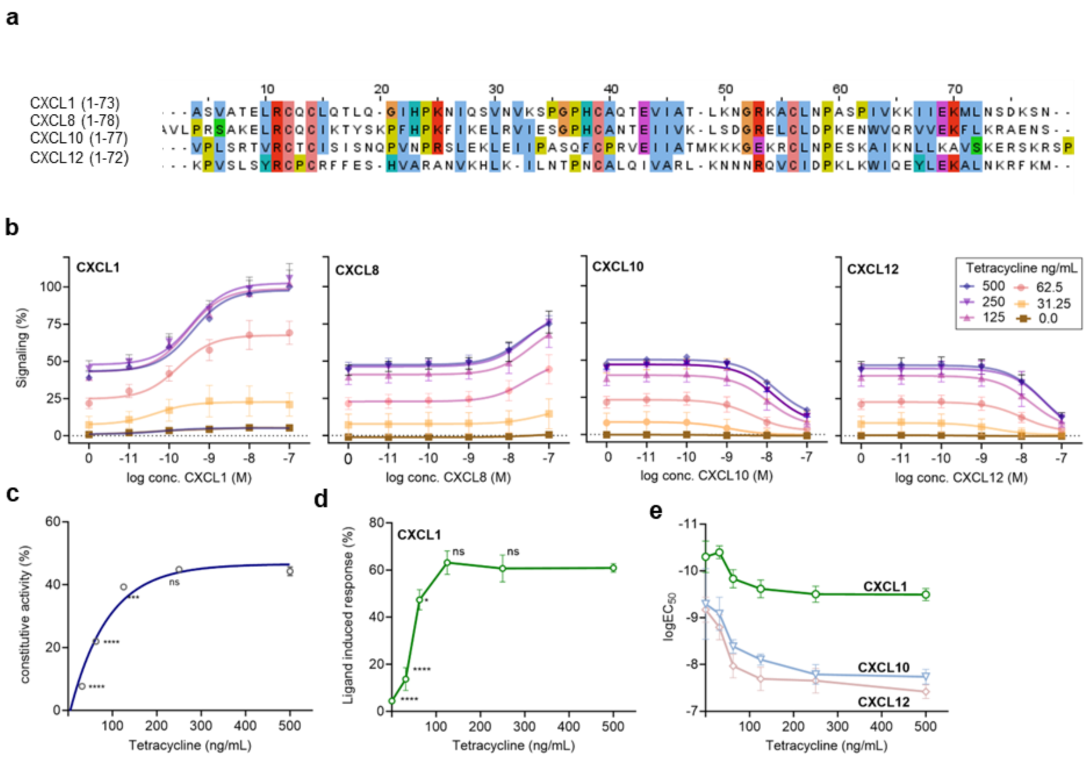

Supplementary Figure 1 – Constitutive and ligand-regulated activity in tetracycline-inducible cells expressing ORF74

a Multiple sequence alignment of chemokine domains of CXCL1 (Uniprot: P09341), CXCL8 (Uniprot: P10145), CXCL10 (Uniprot: P02778), CXCL12 (Uniprot: P48061) using Clustal Omega. Blue: hydrophobic residues (A,C,I,L,M,F,W,V), red: positive charge (K,R), magenta: negative charge (E,D), green: polar (N,Q,S,T), pink: cysteines (C), orange: glycines (G), yellow: prolines (P), cyan: aromatic (H,Y). All sequences are N-terminally processed, i.e. excluding the signal sequences. **b** G_q-signaling measured as inositol triphosphate (IP3) accumulation with the indicated chemokines in stable tetracycline-inducible HEK293 cells expressing ORF74. The tetracycline concentrations (0-500 ng/mL) are shown in the inset in the last panel. Data are normalized to the maximum signal of CXCL1 (%) and represent means ± SEM of *n* = 3 individual experiments performed in duplicates. **c** Constitutive G_q-signaling measured as IP3 accumulation for increasing receptor expression levels induced by increasing tetracycline concentrations. **d** Percent of CXCL1-induced G_q signaling relative to total signaling after induction of ORF74 expression by tetracycline ranging from 125 ng/mL to 500 ng/mL. **e** Cell-killing potency for increasing tetracycline concentrations ($logEC_{50}$ values are provided in Supplementary Table 2). Statistical significances in (c) and (d) were analyzed with one-way ANOVA, by comparison to the value with 500 ng/mL tetracycline. P value: 0.0332 (*), 0.0021 (**), 0.00021 (***), < 0.0001 (****), ns = not significant.

Supplementary Table 1 – Chemokine signaling ($logEC_{50}$) in stable tetracycline-inducible HEK293 cell line expressing ORF74 at various tetracycline concentrations, evaluated by inositol triphosphate (IP3) accumulation in response to stimulation with endogenous chemokines.

	**CXCL1**	**CXCL8**	**CXCL10**	**CXCL12**
Tetracycline (ng/mL)	$logEC_{50}$ ± SEM (n)	$logEC_{50}$ ± SEM (n)	$logEC_{50}$ ± SEM (n)	$logEC_{50}$ ±SEM (n)
0	n/a (4)	n/a (4)	n/a (3)	n/a (4)
31	-10.4 ± 0.1 (2)	n/a (2)	n/a (2)	n/a (2)
63	-9.8 ± 0.2 (4)	n/a (4)	-8.0 ± 0.3 (4)	-8.0 ± 0.3 (4)
125	-9.6 ± 0.2 (4)	n/a (3)	-7.9 ± 0.2 (4)	-7.7 ± 0.3 (4)
250	-9.5 ± 0.2 (4)	n/a (3)	-7.8 ± 0.2 (4)	-7.7 ± 0.3 (3)
500	-9.5 ± 0.1 (4)	n/a (4)	-7.7 ± 0.2 (4)	-7.4 ± 0.1 (4)

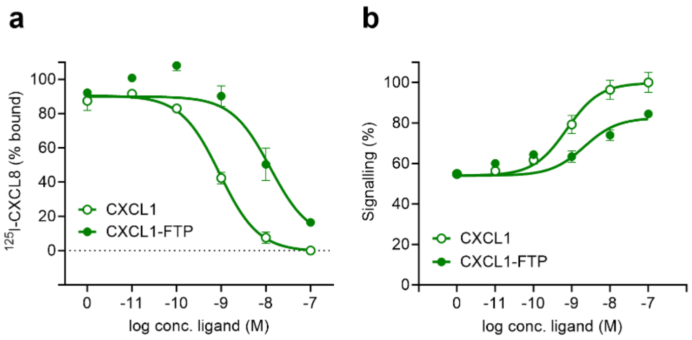

Supplementary Figure 2 - CXCL1-FTP binding and signaling via ORF74

a Binding affinity of CXCL1 and CXCL1-FTP evaluated in competition with ^{125}I-CXCL8 in tetracycline-inducible HEK293 cells expressing ORF74 (induced by 125 ng/mL tetracycline). **b** Inositol triphosphate (IP3) accumulation measured in COS-7 cell transiently expressing ORF74 stimulated with CXCL1 or CXCL1-FTP. Data is normalized to the maximum response of CXCL1 (100 nM). Data represents means ± SEM of n = 3 individual experiments performed in duplicates.

Supplementary Table 2 - Functional data on cell-killing of stable tetracycline-inducible HEK293 cell line expressing ORF74, CXCR1, CXCR2, CXCR3, and CXCR4 (125 ng/mL tetracycline), and HEK293A with the indicated FTPs.

		CXCL1-FTP	CXCL8-FTP	CXCL10-FTP	CXCL12-FTP
**ORF74**	$logEC_{50}$ ± SEM (n)	-11.0 ± 0.1 (5)	-8.2 ± 0.2 (5)	-11.5 ± 0.2 (5)	-10.06 ± 0.2 (5)
	efficacy ± SEM (n)	95 ± 3.4 (5)	85 ± 10.5 (5)	92.4 ± 4.8 (5)	92.5 ± 4.8 (5)
**CXCR1**	$logEC_{50}$ ± SEM (n)	-7.6 ± 0.2 (5)	-9.3 ± 0.2 (3)	-8.5 ± 0.2 (3)	-8.7 ± 0.2 (3)
	efficacy ± SEM (n)	96.7 ± 13 (5)	93.7 ± 6.0 (3)	94.6± 7.0 (3)	73.9 ± 9.6 (3)
**CXCR2**	$logEC_{50}$ ± SEM (n)	-11.2 ± 0.3 (3)	-10.8 ± 0.2 (5)	-8.5 ± 0.2 (3)	-7.7 ± 0.5 (3)
	efficacy ± SEM (n)	98.6 ± 7.01(3)	100 ± 5.71 (5)	100 ± 7.33 (3)	92.1 ± 36.01 (3)
**CXCR3**	$logEC_{50}$ ± SEM (n)	-8.03 ± 0.6 (3)	-7.7 ± 0.5 (3)	-10.1 ± 0.2 (3)	9.03 ±0.2 (3)
	efficacy ± SEM (n)	61.4 ± 21.9 (3)	61.1 ± 21.4 (3)	83.9 ± 7.1 (3)	98.3 ± 6.3 (3)
**CXCR4**	$logEC_{50}$ ± SEM (n)	-7.9 ± 0.4 (3)	-7.8 ± 0.3 (3)	-9.00 ± 0.3 (3)	-11.6 ± 0.3 (5)
	efficacy ± SEM (n)	81.1 ± 15.6 (3)	71.9 ± 13.6 (3)	89 ± 8.8 (3)	88.8 ± 6.00 (5)
**HEK293A**	$logEC_{50}$ ± SEM (n)	-7.8 ± 0,2 (3)	-7.06 ± 0,2 (3)	-8.3 ± 0,2 (3)	-7.6 ± 0,3 (3)
	efficacy ± SEM (n)	85,8 ± 7,9 (3)	74,5 ± 7,4 (3)	100 ± 9 (3)	93,1 ± 29,6 (3)

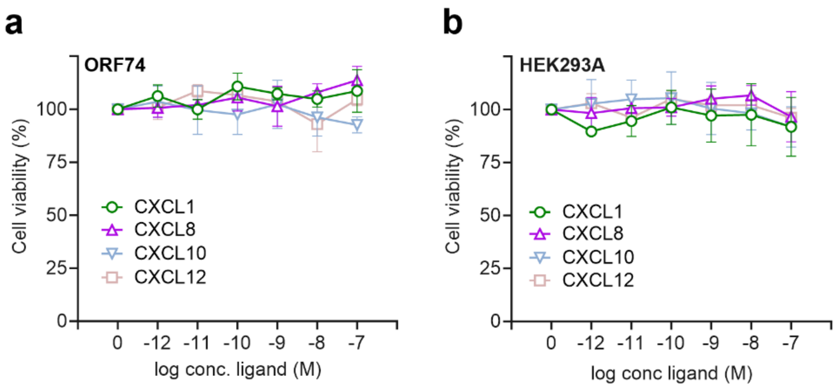

Supplementary Figure 3 - Chemokines alone have no effect on cell viability

a and **b** Cell viability of tetracycline-inducible HEK293 cells expressing ORF74 (at 125 ng/mL tetracycline) (A) and HEK293A cells (B) treated with chemokines. Data is normalized to cycloheximide (100 %) and buffer (0 %) cell-killing, respectively, and represents ± SEM of $n = 4$ individual experiments performed in triplicates.

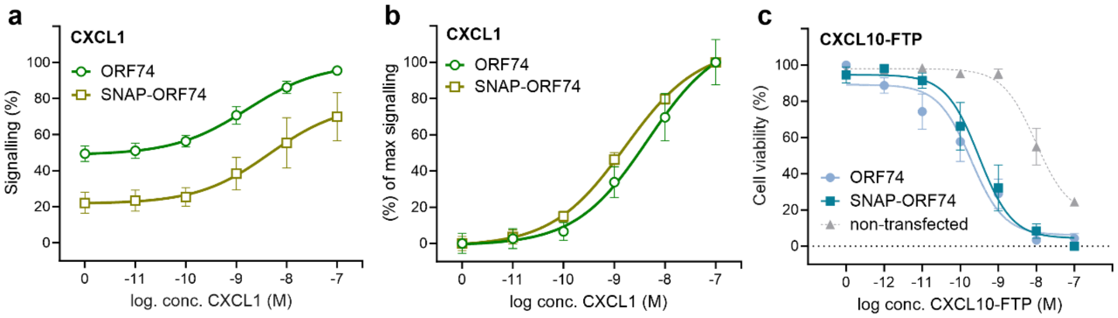

Supplementary Figure 4 – SNAP-tagged ORF74 preserves receptor pharmacology and FTPs mirror chemokine binding and internalization.

a G protein signaling measured as inositol triphosphate (IP3) accumulation in COS-7 cell transiently expressing ORF74 or SNAP-tagged ORF74 and stimulated by CXCL1. Data is normalized to the maximum response achieved by CXCL1 in ORF74-expressing cells. Data represents means and SEM of *n* = 5 individual experiments performed in duplicates. **b** Agonist activation showed as (%) of maximum signaling by CXCL1 based on data from (A) normalized to maximum and minimum response for each receptor. **c** Cell-killing by CXCL10-FTP in transiently transfection of HEK293A cells expressing SNAP-tagged ORF74, ORF74, or in non-transfected cells. Data is normalized to cycloheximide (100 %) and buffer (0 %) cell-killing, respectively. Data represent means ± SEM of *n* = 3 individual experiments performed in triplicates.

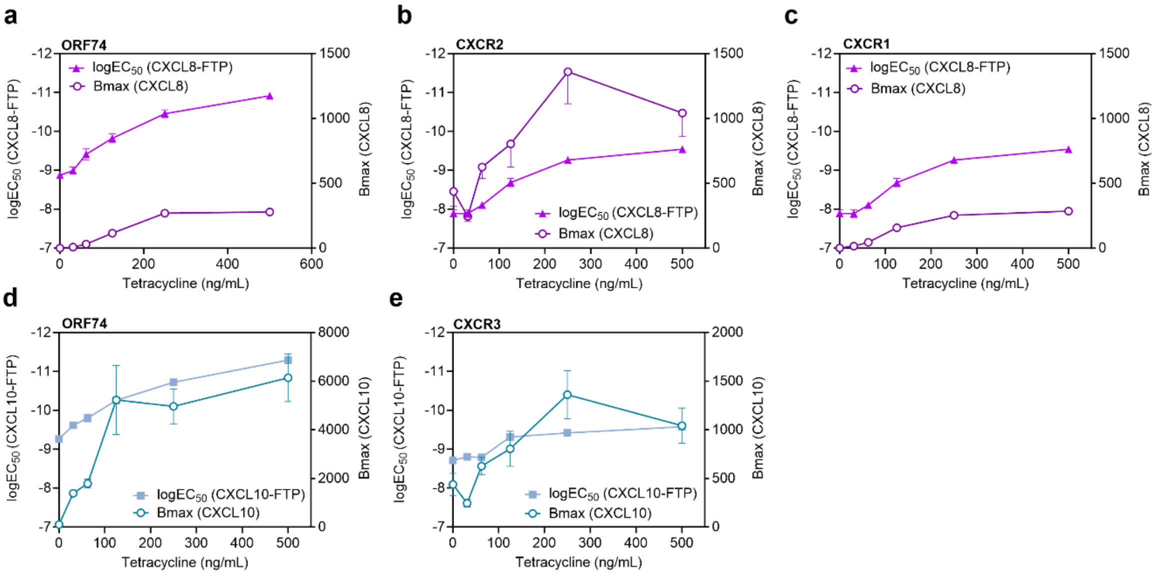

Supplementary Figure 5 – Relationship between cell-killing potency, binding capacity and receptor expression.

a-e Cell-killing potency (logEC_{50}, ± SEM, left Y axis) and maximum binding capacity (B_{max} ± SEM, right y-axis) in tetracycline-inducible HEK293 cells expressing ORF74 and endogenous receptors as a function of increasing tetracycline concentrations (0-500 ng/mL tetracycline). Data represent means ± SEM of *n* = 3 individual experiments performed in either triplicates (killing) or duplicates (binding).

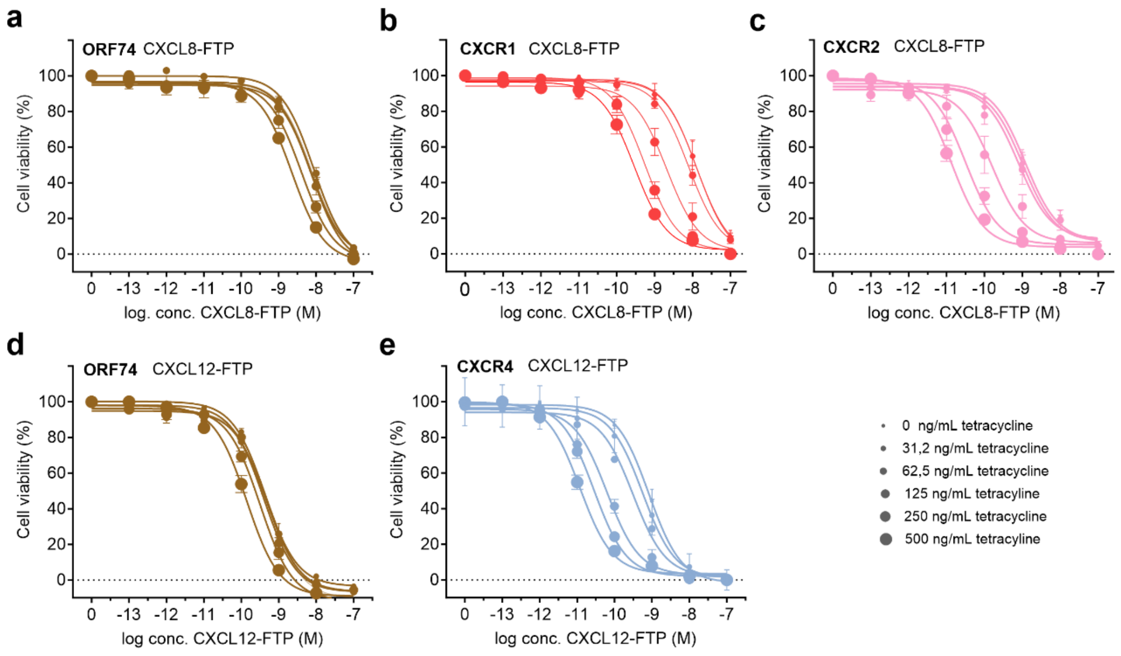

Supplementary Figure 6 – Tetracycline dependent cell-killing

a-e Tetracycline-inducible HEK293 cells expressing ORF74 or the endogenous receptors (CXCR1, 3 or CXCR4), treated with CXCL8-FTP on ORF74 (A), CXCR1 (B), and CXCR2 (C) and CXCL12-FTP on ORF74 (D) and CXCR4 (E). Increasing tetracycline concentration (0-500 ng/mL), represented by dot sizes. Data are normalized to cycloheximide (100 %) and buffer (0 %) cell-killing, respectively, and represent mean ± SEM of *n* = 3 experiments performed in triplicates.

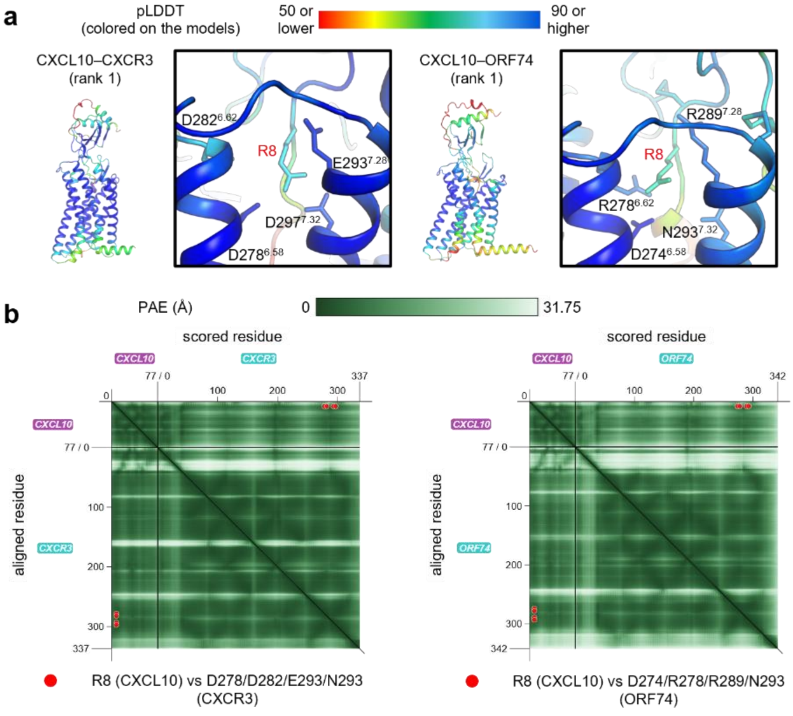

D278	D282	E293	D297
3.42	3.50	2.83	2.98

D274	R278	R289	N293
4.22	4.21	4.08	4.18

Supplementary Figure 7 - Validation of top-ranked prediction of the wild-type CXCR3 or ORF74 complexes with CXCL10.

a pLDDT score colored spectrum on the ribbon model of CXCL10-CXCR3 and CXCL10-ORF74, as indicated in the figure. pLDDT: predicted local distance difference test. **b** PAE plots and values between R8 and predicted R8-binding pocket of CXCR3 or ORF74, indicating 3-4 Å expected errors in relative positions between the residues. PAE: predicted aligned error.

a

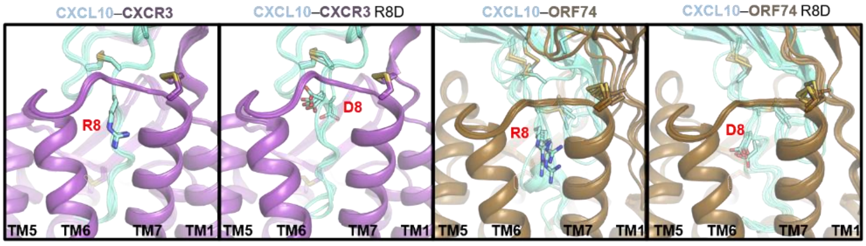

CXCL10–CXCR3

rank	pLDDT	pTM
1	82.4	0.804
2	81.6	0.818
3	81.1	0.799
4	80.4	0.785
5	78.4	0.755

CXCL10–CXCR3 R8D

rank	pLDDT	pTM
1	82.3	0.802
2	81.1	0.813
3	79.9	0.789
4	80.5	0.786
5	78.5	0.758

CXCL10–ORF74

rank	pLDDT	pTM
1	77.5	0.765
2	73.2	0.694
3	73.2	0.723
4	70.8	0.707
5	72.2	0.694

CXCL10–ORF74 R8D

rank	pLDDT	pTM
1	72.9	0.694
2	73.4	0.710
3	71.8	0.712
4	73.1	0.715
5	70.5	0.693

b

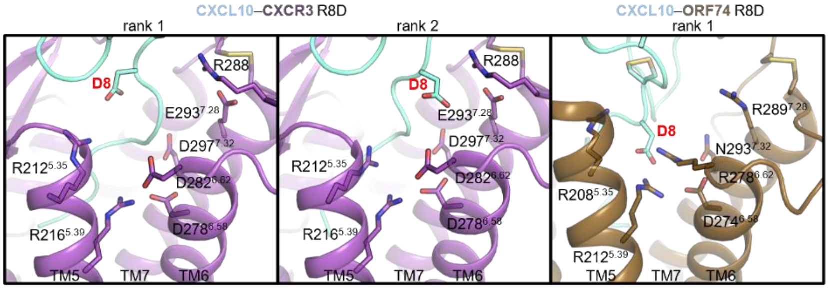

Supplementary Figure 8 - Prediction of binding poses of CXCL10 with CXCR3 or ORF74.

a Five predicted models (rank 1-5) and their statistics for the CXCL10-CXCR3 and CXCL10-ORF74 complexes in the presence or absence of the R8D mutation. The same docking pose and R8 position were predicted for the wild-type CXCL10-CXCR3 complex, although the cryo-EM structure indicates it would be flexible. The R8D mutation makes the sidechain position shallower in the pocket and less reproducible. The opposite trend is observed for the ORF74 complexes. **b** Sidechain interaction of predicted models with the R8D mutation. For CXCR3, two models are displayed because D8 is flipped between the rank 1 and rank 2 structures. The repulsed D8 sidechain might still bind to basic residues surrounding the acidic patch, explaining reduced but maintained potency towards CXCR3 expressing cells together with other interactions at different chemokine recognition sites. ORF74 complexes show similar D8 poses supported by the basic residues in the pocket and nearby TM5. pLDDT: predicted local distance difference test, pTM: predicted template modeling score.
